# Modification and Properties of Cellulose Nonwoven Fabric—Multifunctional Mulching Material for Agricultural Applications

**DOI:** 10.3390/ma14154335

**Published:** 2021-08-03

**Authors:** Tobiasz Gabryś, Beata Fryczkowska, Joanna Grzybowska-Pietras, Dorota Biniaś

**Affiliations:** 1 Department of Materials Science, Faculty of Materials, Civil and Environmental Engineering, University of Bielsko-Biała, ul. Willowa 2, 43-309 Bielsko-Biała, Poland; dbinias@ath.bielsko.pl; 2 Department of Environmental Protection and Engineering, Faculty of Materials, Civil and Environmental Engineering, University of Bielsko-Biała, ul. Willowa 2, 43-309 Bielsko-Biała, Poland; bfryczkowska@ath.bielsko.pl; 3 Department of Civil Engineering, Faculty of Materials, Civil and Environmental Engineering, University of Bielsko-Biała, ul. Willowa 2, 43-309 Bielsko-Biała, Poland; jpietras@ath.bielsko.pl

**Keywords:** viscose, chemical fertilisers, polylactide, degradation, nonwoven crop cover

## Abstract

The paper describes a method of modifying a commercial viscose nonwoven fabric and its use as a modern mulching material in agriculture. The conducted research confirmed that the proposed modification of the viscose nonwoven fabric could be successfully used as a multipurpose and, above all, completely biodegradable nonwoven crop cover, which will eliminate the problem of disposal after the harvest period. Modified cellulose nonwoven fabric was obtained by staining with NB—BT helion brown, then padding with potassium nitrate (KNO_3_) solution (used as a fertilizer) and finally coating with polylactide (PLA) solution. The characterisation of the nonwoven fabric included structural analysis, physicochemical properties and mechanical tests. The modified cellulose nonwovens were used in the tunnel cultivation of tomatoes as a heat-retardant, water-absorbing, antiweed mulching material that prevents soil infestation and slowly releases fertilizers.

## 1. Introduction

The rapidly growing population of people in the world forces food producers to use measures that increase yield and facilitate agrotechnical procedures. In soil cultivation, various types of textile crop covers, ranging from nets, fabrics and nonwovens to films, are playing a very important role. Their main task is to eliminate weed infestation, reduce soil evaporation or protect the soil against erosion and leaching of minerals as a result of heavy rains [1,2,3]. Crop covers are mainly made of synthetic, petroleum-based polymers, such as polyethylene, polypropylene or polyester, which, depending on what is needed from them, are dyed black, white or green [4,5]. The use of agrotextiles in the form of mulching and cover materials brings many benefits, including the elimination of the use of herbicides and mechanical weeding, improvement of soil temperature conditions, the elimination of excessive drying of the soil, better yielding and no contamination of fruit and vegetables by soil [6]. However, their great disadvantage is the fact that they are not biodegradable, and they cannot be recycled once contaminated with soil after the production process [4], generating production waste [7,8].

Therefore, an interest in biodegradable materials began to grow [9]. In recent years, products (including crop covers) made of biopolymers such as poly(lactic acid) (PLA) or poly(butylene succinate) [10] have become very popular. Their properties and relevant uses in agricultural production are strictly determined by the method of their production, the molecular weight of the polymer and the use of processing additives [11]. There are many scientific reports on attempts to use biopolymer composites as mulching and cover materials or for the production of seedling containers that are biodegradable in soil [12]. The period of their degradation ranges from one month to several months, depending on the type of biopolymer and its molecular weight [13]. Most of the aforementioned materials meet the basic assumptions, which are: improvement of plant growth conditions, increased yield and material biodegradability. It is also worth mentioning that nonwovens made of PLA are compostable, but not always biodegradable. These nonwovens are only decomposed by microorganisms under appropriate thermal conditions and at sufficiently high humidity [14].

From an economic point of view, it is most cost-effective to use existing materials (natural waste materials in particular) rather than synthesizing and producing new, biodegradable polymer materials. Crop covers made of textile waste are the subject of great interest [15]. Textile waste is primarily understood as polysaccharide fibres: cotton, linen, hemp, jute and wool (as a protein fibre). These materials are characterized by a diverse chemical structure. The main component of plant fibres is cellulose, while for animal fibres it is keratin. Therefore, fibrous materials of animal origin can be successfully used in the cultivation of green plants, which are characterized by the highest nitrogen demand [16,17]. By the appropriate selection of the amount of plant and animal fibres, it is possible to obtain textile crop covers with unique physicochemical properties and a specified biodegradation time [18]. The speed of the biodegradation processes of fibrous materials is greatly influenced by the degree of their fragmentation. The finely fragmented material has a larger surface on which microorganisms can multiply. Hence, the degradation time can also be controlled by selecting the lengths of the fibres [19,20].

Cellulose materials—such as viscose—are becoming increasingly popular for mulching and the production of plant seedlings. Thanks to their good sorption properties (as well as their quick biodegradation), they are of great interest to researchers. The time of their biodegradation depends on many factors and can range from a few to a dozen or so weeks. Shaari and the team developed a method of producing biodegradable containers from a blend of cotton and viscose fibres that biodegraded over a period of 60 days [21]. Another example of the use of cellulose fibres as a multifunctional mulching material is the nonwovens developed by Ozen’s team. The team developed nonwovens containing potassium nitrate, which had the property of a prolonged release of fertilizer thanks to the use of polyvinyl alcohol [22].

This paper presents the possibility of modifying a commercial viscose nonwoven fabric used in the hygiene industry. The modification consisted in dyeing the nonwoven fabric brown, then soaking it with KNO_3_ solution, and subsequently coating it with PLA solution. As a result of these modifications, a mulching material that provides the cultivated plants with optimal thermal and water conditions and protection against weed infestation was obtained. An interesting solution of the modified nonwoven fabric was the application of a PLA coating, which firstly slowed down the release of mineral fertilizer (KNO_3_) into the growing substrate. Secondly, PLA slowed down the degradation process of cellulose as the base material of the nonwoven, which extended the process of its use in cultivation to 15 weeks.

## 2. Experimental

### 2.1. Materials

Viscose nonwoven fabric obtained using the spunlace method with a surface mass of 55 g/m^2^ (LENTEX S.A., Lubliniec, Poland), polylactide (3050, Nature Works LLC, Blair, NE, USA), KNO_3_, Na_2_CO_3_, NaCl and CHCl_3_ (99.8%) (Avantor Performance Materials Poland S.A., Gliwice, Poland, NB-BT helionic brown dye (Boruta-Zachem S.A., Zgierz, Poland), COMPO BIO universal potting soil (COMPO GmbH, Munster, Germany).

### 2.2. Modification of Cellulose Non-Woven Material

Our previous research on the production of long-acting cellulose fertilizer granules [23] provided us with the inspiration for the modification of cellulose nonwoven material. At the beginning, the possibility of using unmodified viscose nonwoven as a mulching material in tomato greenhouse cultivation was investigated. Field studies have shown that the degradation time of pure cellulose is only 1 month; therefore, it was modified.

**Figure 1:**
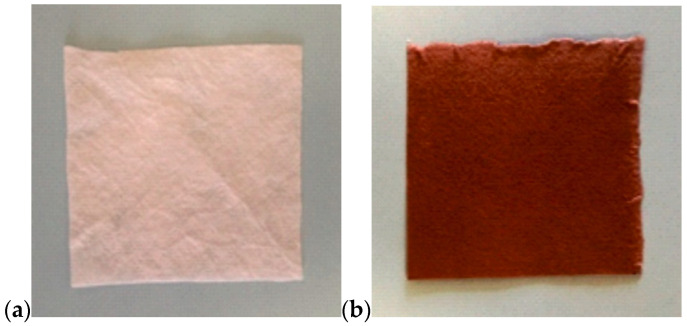
Photograph of the nonwoven (**a**) before the dyeing process; (**b**) dyed nonwoven.

The viscose nonwoven fabric was first dyed in order to provide plants with better thermal conditions in the soil by absorbing solar radiation. The second reason for dyeing was to limit sunlight getting to the soil layer beneath the nonwoven layer to eliminate weed growth. The viscose nonwoven fabric (Figure 1a) was dyed in accordance with the BN-86 6041-19 standard [24], using NB-BT helion brown as a colouring agent. The cellulose fibres dyeing process was carried out at 80 °C. Initially, the material to be tested was introduced into the dyeing bath with 2% dye (in relation to the weight of the nonwoven) and dyed for 30 min. After this time, a portion of Na_2_CO_3_ was added to the dyeing bath (30 g/dm^3^ of the bath), and after 10 min another portion of Na_2_CO_3_ (30 g/dm^3^ of the bath) was added. The dyeing continued for a further 30 min, after which 4 g/dm^3^ of NaCl of the bath was added at the end of the dyeing process, The dyed nonwoven was rinsed and dried (Figure 1b).

The next step was to carry out the modification of the dyed nonwoven. Table 1 summarizes the designations of the modified nonwovens.

The first modification consisted in covering the viscose nonwoven with a thin layer of polylactide (PLA) in order to slow down the cellulose biodegradation process. Initially, a 1% solution of PLA in chloroform (CHCl_3_) was prepared, which was sprayed on the nonwoven and then dried in an oven at 80 °C (Table 1, nonwoven A). The amount of the applied solution was 100 cm^3^/m^2^ of the nonwoven.

The second modification consisted in soaking the viscose nonwoven with potassium nitrate (KNO_3_) in order to give it fertilizing properties. The nonwoven was introduced into a vessel containing a 10% aqueous solution of KNO_3_, then pressed and dried in an oven at 80 °C (Table 1, nonwoven B).

**Figure 2:**
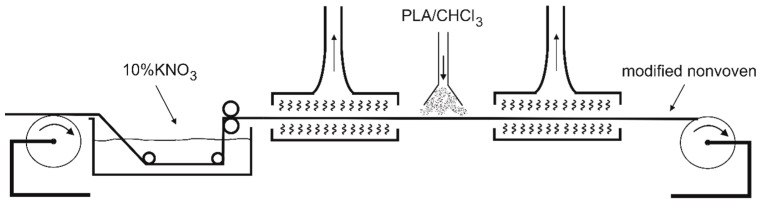
Diagram of the viscose nonwoven C modification process.

The third modification was a combination of the two previous ones, whereby the cellulose nonwoven was first soaked with a 10% KNO_3_ solution, then pressed and dried at 80 °C, and then sprayed with 1% PLA solution in CHCl_3_ and dried again at 80 °C (Table 1, nonwoven C). The viscose nonwoven obtained in this way (Figure 2) was used for field research as a heat-retardant, biodegradable mulching material that gradually releases mineral salts.

### 2.3. Properties of Nonwovens

In order to determine the properties of the nonwovens obtained in the experiment (samples: 0; A; B; C), the following tests were carried out: surface mass, air permeability, perpendicular water permeability, breaking force and relative elongation.

In the case of nonwoven C, which was selected and used in the 15-week cycle of tomato cultivation, every two weeks, the appropriate number of samples for testing was taken, air-dried, and then soil remnants and other contaminants were removed using compressed air. The samples purified in this way were subjected to the same tests as described above [25].

First, the surface mass of the nonwovens was determined. For this purpose, samples with dimensions of 15 × 15 cm were weighed using SARTORIUS CP224S-0CE analytical balance with an accuracy of 0.0001 g. The surface mass (Ws) of the nonwovens (g/m^2^) was calculated according to the following formula:

$$W_{s}=\frac{w}{s}$$

where: *w* = mass of the nonwoven sample (g) and *s* = surface area of the nonwoven (m^2^).

Air permeability was tested using the FX 3300 Air Permeability Tester measuring equipment in accordance with PN-EN ISO 9237:1998 [26]. The measurement was carried out in a clamp with a surface of 20 cm^2^, with a pressure difference of ΔP = 200 Pa. Ten measurements were made for each sample.

The measurement of perpendicular water permeability was determined on the basis of the PN-EN ISO 11058 standard entitled “Geotextiles and geotextile-related products. Determination of water permeability characteristics normal to the plane, without load” [27]. Material samples with a diameter of 60 mm were placed in the measuring equipment and then tested. Ten measurements were made for each sample.

The breaking force and relative elongation tests of nonwovens were carried out according to the procedure described in the PN-EN ISO 13934-1: 2013 standard “Textiles. Tensile properties of fabrics. Part 1: Determination of maximum force and elongation at maximum force using the strip method” [28]. Ten samples with a width of 5 cm and a length of 15 cm were cut from the tested nonwovens. The samples were acclimatized under normal climate conditions at a humidity of 65 ± 2% and a temperature of 20 ± 2 °C. Then, measurements of the breaking force were carried out using the INSTRON model 4554 testing machine. Measurements were carried out at 20 mm/min for samples clamped in the 100 mm machine holders. The breaking force in cN and the relative elongation at break in % were recorded on the stress-strain curve for the samples.

The rate of KNO_3_ release from modified nonwovens was tested in two ways: (1) by examining the desorption of salts into water; (2) by examining the desorption of salts into the soil. The salt desorption of nonwovens into water was tested via the conductometric method using a METTLER TOLEDO SG7 conductometer. Two nonwovens were selected for the tests: B and C, from which samples with a diameter of 10 cm were cut. The samples were placed in separate beakers containing 250 cm^3^ of distilled water and the conductivity of the solution was measured every 5 min. For nonwoven C, for which the desorption time was long, the test time was extended and additional measurements were carried out after 24, 48, 72 and 96 h.

The rate of KNO_3_ release into the soil, on the other hand, was investigated by measuring the potassium content with the spectrometric method using the Perkin Elmer Atomic Absorption Spectrometry Analist 100 atomic absorption spectrometer (AAS). For these tests, nonwoven C was selected, from which samples with a diameter of 10 cm were cut. Batches of 120 g of potting soil with a humidity of 80% were introduced into glass crystallizers with a diameter of 12 cm, and then the previously prepared samples of nonwovens were placed on the top layer. The tests were carried out for 4 weeks on four series of samples. Soil moisture was maintained at 80% and monitored with a soil moisture meter. Subsequent series of tests were terminated after 1, 2, 3 and 4 weeks. For this purpose, the nonwoven was removed from the substrate, and 250 cm^3^ of distilled water was introduced into the crystallizer with the soil and thoroughly mixed. Then, the solution was decanted and filtered on the analytical funnel, following which the potassium concentration in the subsequent samples was determined with the use of AAS. In addition, the potassium content in the clean soil used for the test was determined in the same way as described above.

### 2.4. The Use of Modified Nonwovens as Mulching Material

**Figure 3:**
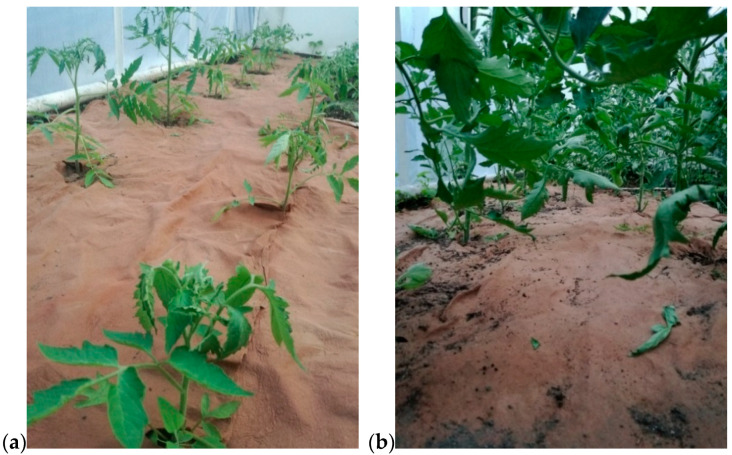
Mulching the tomatoes with nonwoven C: (**a**) at the beginning of the research, (**b**) after 2 months of the research.

Nonwoven C, modified by padding with KNO_3_ and then coating with PLA, was used for field tests (Figure 2). The nonwoven was used as a mulching material in the greenhouse cultivation of the tomatoes. It was placed on the surface of the soil and secured to the substrate with metal pins. Then, incisions were made in the nonwoven, through which the plants were then planted. Standard soil care was applied throughout the tomato cultivation period. Every 2 weeks, small samples of the nonwoven were collected in order to evaluate its physicochemical and structural properties. The photo of plant cultivation with the nonwoven C is presented in Figure 3.

### 2.5. Structural Analysis

Nonwoven tests were also conducted using a high-resolution Phenom ProX scanning electron microscope (SEM) from Thermo Fisher Scientific (Pik Instruments, Piaseczno, Poland) that was operated at 10 kV. The samples were previously coated with a 10 nm gold layer using a Leica EM ACE 200 low-vacuum coater.

Wide-angle X-ray scattering (WAXS) studies were performed using a URD-65 Seifert diffractometer (Germany) using the Bragg–Brentano reflection geometry method. CuKα radiation (λ = 1.54 Å) was emitted at an accelerating voltage of 40 kV and an anode current of 30 mA. The monochromatization of the radiation beam was achieved by placing a graphite monochromatizer across the monochromatized beam. A scintillation counter was used as a detector. The tests were carried out in the range of 2θ from 3° to 60° in steps of 0.1°. Before the measurements, the nonwovens were thoroughly rinsed to remove salt residues and earth particles, then dried and pulverized with a microtome.

Nicolet 6700 FT-IR spectrometer (Thermo Electron Corp., Madison, WI, USA) equipped with a diffuse reflectance accessory EasiDiff (PIKE Technologies, Inc., Madison, WI, USA) were used in the FTIR spectroscopic analysis. The following measurement parameters were used: resolution, 8 cm^−1^; spectral range, 4000–500 cm^−1^; and number of scans, 64. Data collection and post-processing were conducted using the OMNIC software (v. 9.0, ThermoElectron Corp., Madison, WI, USA.).

The spectrometer MAGNA-IR 860 and NICOLET with a FT-Raman accessory were used to record the Raman spectra of the samples. The solid samples were then irradiated with a 1064 nm line YAG laser and scattered radiation were collected with 8 cm^−1^ resolution.

## 3. Results and Discussion

### 3.1. General Characteristics of the Modified Nonwovens

In order to determine the physicochemical properties of modified cellulose nonwovens, parameters such as surface mass, air permeability and perpendicular water permeability, breaking force and relative elongation were tested, and the obtained results are summarized in Table 2.

At the beginning, the influence of individual modifications on the surface mass of the obtained nonwovens was analysed. Studies have shown that 1% PLA solution coating slightly increases the surface mass of nonwoven A to 55.6 g/m^2^ (i.e., 0.8% more than the surface mass of the nonwoven 0). On the other hand, soaking the material with 10% KNO_3_ solution increased the surface mass of nonwoven B by ~120%. This indicates a high sorption of salt solution, which significantly increased the weight of nonwoven B. In the case of nonwoven C, it was observed that the presence of KNO_3_ crystals, which were initially applied on the nonwoven, made the PLA layer larger and the mass uptake of the PLA increase to 4%. By that means, total mass uptake for nonwoven C is about 130% higher compared to nonwoven 0.

When analysing the air permeability results (Table 2), it can be seen that the PLA coating slightly sealed the spaces between the individual fibres in the nonwoven A. As a result, the air permeability values for nonwoven A were 2443 dm^3^/m^2^/s. On the other hand, the introduction of KNO_3_ on the nonwoven B resulted in an approximately 12% decrease in air permeability as compared to the original nonwoven (nonwoven 0). Air permeability for sample C modified with salt and PLA was comparable to that of nonwoven 0 and amounted to 2758 dm^3^/m^2^/s. These results demonstrated that the use of nonwoven C as a mulching material in agriculture will not block the access of atmospheric air to the deeper layers of soil.

In perpendicular water permeability tests (Table 2) for nonwoven A, about a 45% decrease as compared to the original nonwoven 0 can be observed. The value could result from the presence of PLA on the surface of the nonwoven, making it hydrophobic and preventing water from penetrating deep into the nonwoven. Moreover, PLA covering KNO_3_ crystals also slightly hydrophobise the surface of nonwoven C. As a result, the perpendicular water permeability decreased from 77 × 10^−3^ (m/s) for nonwoven B to 72 × 10^−3^ (m/s) for nonwoven C. These results clearly indicate the water barrier function of the PLA coating, which reduced the perpendicular water permeability of modified nonwovens A and C.

Analysing the breaking force values, it can be noticed that all the proposed modifications to the cellulose nonwoven resulted in the increase of the breaking force (Table 2). Coating nonwoven fabric A with PLA solution increased the breaking strength by 53%, but significantly reduced the relative elongation to ~3.7 (mm). Thus, the modification increased the strength and brittleness of nonwoven A, while in the case of nonwovens modified with KNO_3_ (nonwovens B and C), an increase in the breaking force and a slight decrease in relative elongation were observed. This phenomenon is likely to be related to the formation of weak interactions between viscose fibres and salt, which additionally strengthens the nonwovens, only slightly deteriorating their elongation. This relationship consists only of the physical consolidation of the material, which increases its strength.

**Figure 4:**
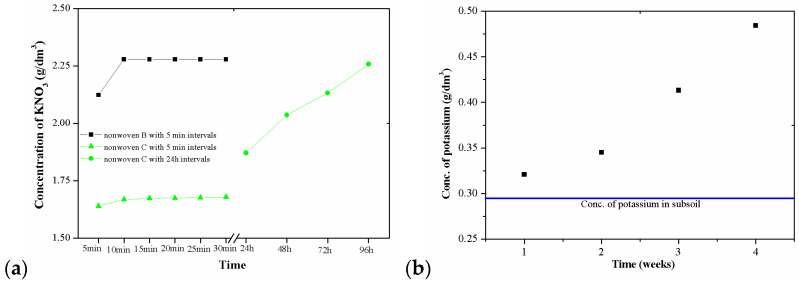
Kinetics of KNO_3_ release: (**a**) from nonwovens B and C to water; (**b**) from nonwoven C to the soil.

An important feature proving the possibility of using modified nonwoven crop cover as carriers of slow-release fertilizers is the release kinetics of the salt used in the experiment. The tests of KNO_3_ desorption to water performed initially (Figure 4a) demonstrated that nonwoven B released the salt applied in the modification process very easily and quickly. After 5 min of testing, the KNO_3_ concentration of nonwoven B was 2.12 (g/dm^3^), and after 10 min it stabilized at the level of 2.28 (g/dm^3^). Nonwoven C, in contrast to nonwoven B, is characterized by a much slower desorption of salt. This is very beneficial and improves the possibility of using this material in agriculture. In this case, the concentration of the aqueous solution from the beginning of the measurement was 1.64 (g/dm^3^) and reached the value of ~1.68 (g/dm^3^) within 30 min. The following days of KNO_3_ desorption tests of nonwoven C indicated that only after 4 days did the salt concentration reach 2.26 (g/dm^3^). The desorption tests confirmed that the PLA layer (which covered nonwoven C) effectively protects the salt that is well-soluble in water against quick washing out from the cellulose nonwoven. It can be assumed that at the beginning of the process, KNO_3_ is desorbed from those fragments of nonwoven C that were not covered with the PLA layer. The washing out of successive portions of salt can take place in two ways: either by slow rinsing out through the pores in the PLA coating or due to PLA degradation.

The desorption of KNO_3_ from nonwoven C to the soil was much slower (Figure 4b) compared to the desorption into the water. The initial potassium concentration in the soil was 0.295 (g/dm^3^). In the next 4 weeks of the study, the concentration of potassium in the tested soil slowly increased and amounted to 0.32; 0.35; 0.41; 0.48 (g/dm^3^), respectively. The studies indicated that KNO_3_ slowly desorbed into the soil; however, the amount of desorbed salt was not constant, but slowly increased over time. The gradual desorption of KNO_3_ is favourable because it provides ever greater doses of potassium (which is greatly needed in tomato cultivation). Moreover, it can also be assumed that the release of KNO_3_ from nonwoven C into the soil takes place in a similar manner as described above for the desorption of salt into water. Salt desorption is accompanied by a slow degradation of the nonwoven C structure, which can be seen in Figure 3b.

### 3.2. Analysis of Nonwoven Biodegradation Process

During field tests, samples of nonwoven C were collected in order to assess the degree of its degradation. Changes in the structure of the nonwoven fabric were noticeable to the naked eye, but the actual image of degradation was obtained using a scanning electron microscope (Table 3).

When analysing the photographs (Table 3 (a)), the different stages of material decomposition can be noticed. In the first 2 weeks, a KNO_3_ deposit appeared on the surface of the nonwoven (sample C2a). Formation of the so-called “salt efflorescence” is a natural phenomenon that occurs when the mulch material comes into contact with moist soil. The initial stages of the PLA distribution, which are visible as the degradation of the polymer film on the surface of the fibres, can also be seen (sample C2c). A similar process is described by Sharma et al. in their paper, where they studied the biodegradability of the PLA films in the natural environment [29]. Then, wrinkles appeared on the surface of nonwoven (samples C4a to C8a). In the photograph of sample C10a, the beginning of the degradation of the nonwoven structure can be observed, which is clearly visible in the photograph of sample C12a. Thus, approximately 10 weeks after the application of nonwoven C, the process of the degradation of cellulose fibres, which form the base of the mulching material, began.

**Figure 5:**
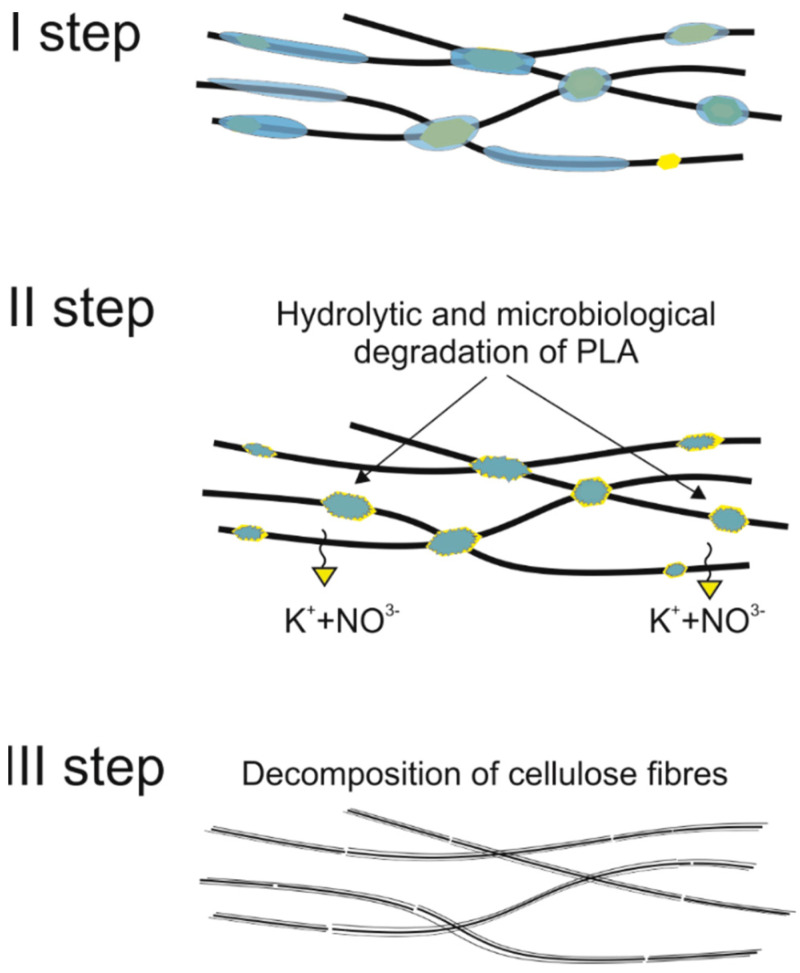
Stages of nonwoven C degradation.

Even more information can be obtained by analysing SEM micrographs of nonwoven C samples (Table 3 (b); Table 3 (c)). In the SEM pictures of the original nonwoven fabric (samples 0b and 0c), it can be observed that the cellulose fibres are covered with KNO_3_ crystals in the form of thin needles (Figure 5—I step). While on the samples of nonwovens obtained after 2 weeks of testing (samples C2b and C2c), there are no needles of salt, but a porous PLA film can be observed (sample C2c). PLA, after fulfilling its role as a hydrophobic layer on salt crystals, decomposes into lactic acid, which additionally slightly acidifies the soil, creating optimal conditions for plant development. The analysis of subsequent micrographs, starting from the sample C4 to C12, showed that in the following weeks of study salt desorption, combined with PLA degradation, took place (Figure 5—II step). It can also be assumed that the degradation of PLA took place both under the influence of microorganisms present in the soil and as a result of the hydrolysis of the polymer itself. The photos of the C12b and C12c samples also show a clear breakdown of individual cellulose fibres forming the structure of nonwoven C (Figure 5—III step) into fibrils.

The degradation process of the modified nonwoven C was also investigated by changing such physicochemical parameters as surface mass, air permeability, perpendicular water permeability, breaking force and relative elongation. The results are summarized in Table 4.

When analysing the surface mass results (Table 4), it could be noticed that after 4 weeks, 39% of nonwoven C4 was lost, which is consistent with the observations made with the use of SEM (Table 3). In accordance with the KNO_3_ desorption studies described earlier, during this period salt was released mainly from the spaces that were not covered with the PLA layer (Figure 4b). This resulted in a decrease in air permeability to the value of ~2486 (dm^3^/m^2^/s), with a simultaneous increase in water permeability to ~76 × 10^3^ (m/s).

The following 6 weeks of studying nonwoven C were the time of salt desorption and PLA degradation taking place simultaneously (Figure 5—II step). The mass of nonwoven C10 decreased by another 20%, the value of air permeability decreased to ~1996 (dm^3^/m^2^/s). Comparing the obtained results to the SEM image of nonwoven C10 samples (Table 3), it can be said that the defibreing of the structure of individual cellulose fibres in the nonwoven may result in air flow resistance and an increase in the perpendicular water permeability up to ~77 × 10^3^ (m/s).

Further degradation of the studied nonwoven C (Table 4) leads to a further reduction of the initial surface mass of the nonwoven C12 to ~33 (g/m^2^), which is 60% of the mass of nonwoven 0. After 12 weeks of testing, the air permeability values dropped to the lowest level as compared to all tested samples, and the water permeability increased slightly.

In the last week of field tests, nonwoven C samples were degraded in 88% (Figure 5—III step). It could be seen with the naked eye that the nonwoven fabric is very thin and has multiple holes. As a result of degradation, there was an increase in the values of air permeability (~2965 (dm^3^/m^2^/s)) and perpendicular water permeability (~178 × 10^3^ (m/s)).

When analysing the breaking force results (Table 4), it was observed that after 4 weeks of testing the strength parameters dropped by 30%. While the following weeks of field tests resulted in a sharp drop in the breaking force to the value of ~187 (cN) for the C14 sample.

The last studied parameter was the relative elongation, which increased slightly during the initial 10 weeks of the study (~8.8 (mm)), and then decreased to the initial value determined for sample C (Table 2). This phenomenon can be explained by the rinsing of KNO_3_ from the interfibre space, followed by the degradation of PLA, which resulted in the loosening of the nonwoven structure with positive effect on elongation. After 14 weeks of study, nonwoven C14 was so badly degraded that the relative elongation took the value of ~10 (mm).

### 3.3. Analysis of Structural Changes Taking Place in the Tested Material

X-ray structure studies (WAXS) and infrared spectroscopy (FTIR and Raman) were used to analyse changes in the structure of modified viscose nonwovens.

The degree of crystallinity of nonwovens was determined on the basis of WAXS analysis. For this purpose, each WAXS pattern was distributed into individual crystalline and amorphous components using the WaxsFit software [30]. This software was used to calculate the degree of crystallinity and the size of the crystallites. In this program, the decomposition is performed by means of an approximation method. It consists in construction of a theoretical curve, which is composed of functions related to individual crystalline peaks and amorphous maxima. The shape of each peak was approximated using linear combination of the Gaussian and Cauchy’s functions. The parameters of these functions are found through best fitting of the theoretical curve to the experimental one using a suitable optimization procedure. The theoretical curve was fitted to the experimental data using the hybrid system 1 optimization method, which is described by Rabiej et al. [31]. The degree of crystallinity was determined as the ratio of the sum of the surface areas under the crystalline peaks to the total area under the scattering curve.

**Figure 6:**
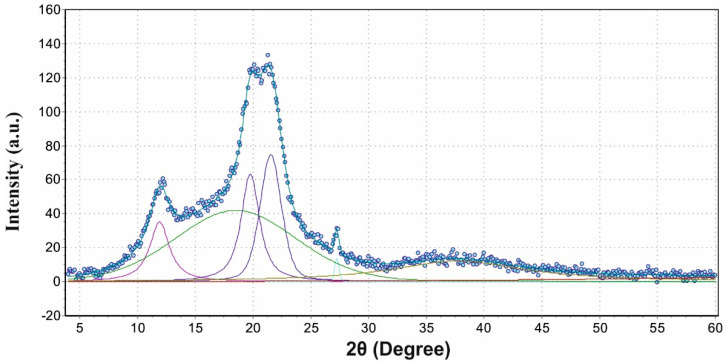
Distribution of the diffraction pattern into crystalline and amorphous components on the example of sample 0 (original viscose nonwoven).

The analysed diffraction patterns are characteristic for the examined material, i.e., viscose. The crystal structure of all samples is typical for type II cellulose, the so-called regenerated cellulose. An example of a diffraction pattern and its distribution into crystalline and amorphous components is presented in Figure 6.

The analysis of data determined on the basis of diffraction patterns allowed to determine the degree of crystallinity, which is an important parameter defining the degradation of the material. The original material (sample 0) is characterized by the degree of crystallinity of 38%, which is a standard value for viscose materials [32]. The degree of crystallinity was not determined for nonwoven C, which underwent a multi-stage modification process. The reason was simple: the presence of KNO_3_ results in the formation of numerous reflections, which makes the correct distribution of the diffraction pattern difficult to obtain and makes it impossible to establish the actual degree of crystallinity value. Subsequent samples, from C4 to C14, indicated a gradual increase in the degree of crystallinity, successively amounting to 41%; 42.2%; 43.9% and 47.2%. During the biodegradation of the material, the amorphous areas, being less ordered and more loosely packed, decomposed first and provided a source of nutrients for microorganisms. Due to enzymatic degradation, they were decomposed first. The pouched dependence showed in his work Puchalski et al. [33], where he described the increase in the degree of crystallinity of PBS along with the time of staying in the compost. Therefore, the total size of the amorphous areas decreased. Crystalline regions, being more tightly packed, were less susceptible to attack by microorganisms. As a result, these regions begin to dominate the overall structure of viscose, as confirmed by the degree of crystallinity values. In addition, the increase in the degree of crystallinity during the biodegradation of the material allows you to better understand the other macroscopic changes in the material. A higher degree of crystallinity after biodegradation favours the fragmentation of the material (which has been shown in strength studies [13,33]). The average size of crystallites, on the other hand, did not change significantly and ranged from 43–45.1 (nm). This is due to the fact that it was mainly amorphous areas that were degraded, which did not have a direct impact on the size of the crystallites in the crystal stacks.

**Figure 7:**
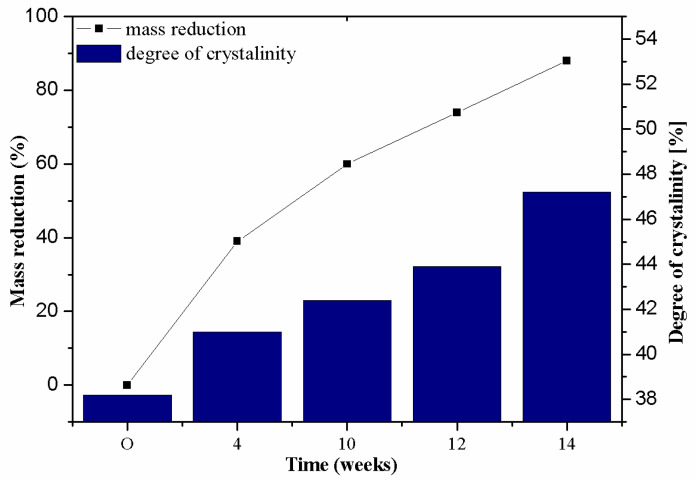
Dependence of the material mass loss over time on the degree of crystallinity.

The fact that the biodegradation process of nonwoven C occurred in stages is also indicated by mass loss (Table 4, Figure 7). In the first 4 weeks of the study, the sample mass decreased by ~39%, which is caused by the release of KNO_3_ which constitutes about 54% of the total mass of nonwoven C. Up to the 10th week of the study, another 20% of the nonwoven was lost (Figure 5—II step). During this time, it simultaneously desorbed salt and degraded PLA. Finally, the cellulose fibres were defragmented (Figure 5—III step), which loosened the structure of the mulching nonwoven. During 12, and then 14, weeks of testing, the mass loss of the nonwoven was 74 and 88%, respectively. The period of biodegradation ending in the 15th week of study is sufficient to complete the yielding of plants and start agrotechnical treatments.

**Figure 8:**
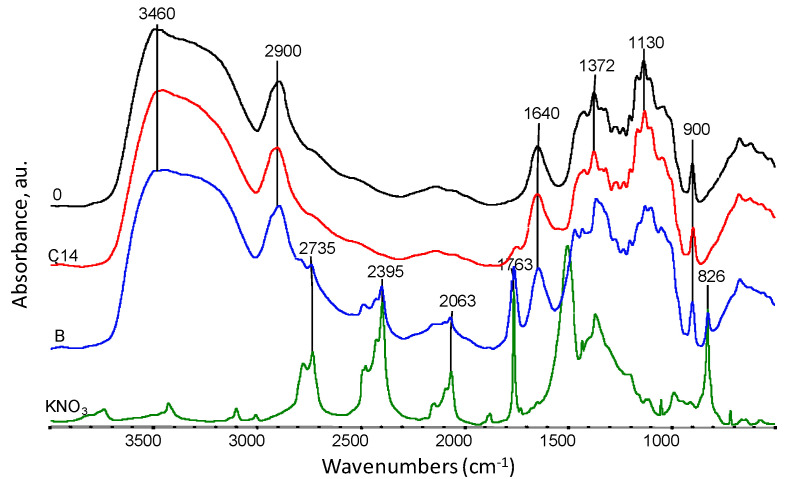
Summary of FTIR spectra for samples: 0—viscose nonwoven, B—KNO_3_ modified nonwoven, C14—modified nonwoven C after 14 weeks of study, and KNO_3_ salt spectrum at 4000–400 cm^−1^.

The chemical structure of viscose nonwovens (sample 0) and KNO_3_-modified nonwovens (nonwoven B and C) was also investigated using FTIR and Raman spectroscopy (Figure 8). The spectra of all studied nonwovens showed a characteristic broad absorption band of O-H group vibrations at wave numbers in the range from approximately 3700–3000 cm^−1^ [34,35]. The position of this band is influenced by intermolecular hydrogen bonds. Weaker hydrogen bonds move the band maximum towards higher wavenumbers, while stronger hydrogen bonds move the band maximum towards lower wavenumbers. The spectra showed a characteristic absorption band at 2900 cm^−1^, which is the result of radiation absorption by C-H groups [34,35]. For samples 0, B and C14, slight changes between 1130–1043 cm^−1^ were observed. This range is related to the C-O-C group vibrations occurring in the glucopyranose ring and in ether bridges [36] and indicates a partial degradation of cellulose. On the FTIR spectrum obtained for nonwoven B, the appearance of characteristic absorption bands occurring in the salt spectrum (KNO_3_) can be observed at 2735 cm^−1^, 2395 cm^−1^, 2063 cm^−1^ and 1763 cm^−1^, which can be attributed to vibration overtone bands of -N=O groups observed in the salt spectrum. After 14 weeks of degradation (sample C14), the salt bands disappeared, which may indicate their complete removal from nonwoven C.

**Figure 9:**
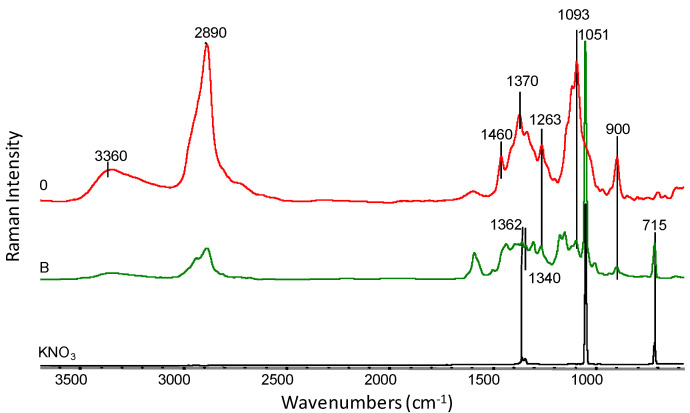
FT-Raman spectra in the range of 3700–500 cm^−1^ for: 0—viscose nonwoven, B—KNO_3_-modified nonwoven and pure potassium nitrate crystals (KNO_3_).

On the FT-Raman spectrum (Figure 9) for a sample of nonwoven modified with KNO_3_ (sample B), all characteristic absorption bands appearing in the salt spectrum were observed: 1362 cm^−1^, 1340 cm^−1^, 1051 cm^−1^ and 715 cm^−1^. Moreover, a strong influence of salt on the characteristic absorption bands present in pure viscose (sample 0) is evident. The reduction of sharp bands at the maximum of wavenumbers 1370 cm^−1^ and 1093 cm^−1^ [36,37], which are characteristic for the C-O-C groups in oxygen bridges, proves that the cellulose structure is the loosened and swollen as a result of salt sorption.

**Figure 10:**
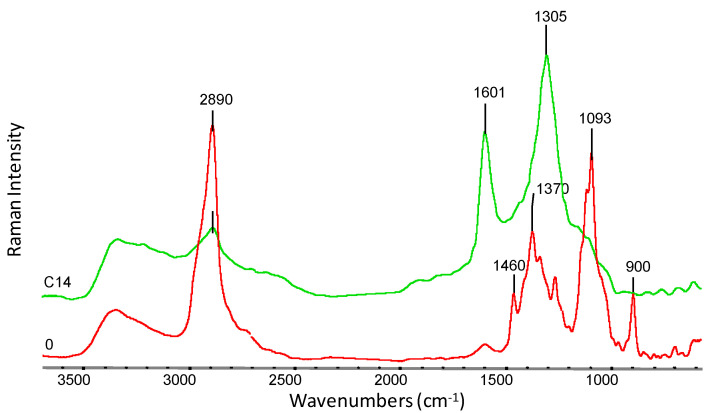
FT-Raman spectra in the range of 3700–500 cm^−1^ for: 0—viscose nonwoven, C14—modified nonwoven C after 14 weeks of study.

The FT-Raman spectrum (Figure 10) for nonwoven C after 14 weeks of testing (sample C14), on the other hand, presents the disappearance of the absorption bands characteristic for the original viscose spectrum (sample 0) in the area of 1460–900 cm^−1^ [36,37]. On the C14 curve, there are two distinct absorption maxima at 1601 cm^−1^ and 1305 cm^−1^. The appearance of these bands may be the result of changes in the viscose structure during the polysaccharide chain degradation process. Shortening the macromolecule chain promotes an increase in the ordering in the material, which in turn affects the shape and shifts of the emission bands in Raman spectra.

## 4. Conclusions

The paper describes a method of modification of a commercial viscose nonwoven fabric manufactured by LENTEX for applications in the hygiene industry. The choice of viscose for the study was dictated primarily by the easy degradability of this material. The modification of the nonwoven was carried out in the following stages: (1) dyeing with NB—BT helion brown; (2) padding with a solution of potassium nitrate (KNO_3_); (3) coating with a polylactide (PLA) solution. The obtained material had the following characteristics: surface mass ~127 (g/m^2^), air permeability ~2760 (dm^3^/m^2^/s), perpendicular water permeability ~72 × 10^3^ (m/s), breaking force ~1148 (CN) and relative elongation ~7.8 (mm). The modified nonwoven C prepared in this way was used as a mulching material in a 15-week-long tomato cultivation. The physicochemical and structural studies conducted in the following weeks made it possible to observe the process of degradation. Salt desorption and scanning electron microscopy (SEM) studies have made it possible to conclude that salt desorption occurs in the first weeks of using the mulching nonwoven. Then two parallel phenomena occur: the desorption of KNO_3_ coupled with the degradation of PLA. At the same time, wide angle X-ray scattering (WAXS) studies demonstrated that the degree of crystallinity of cellulose increases to ~44% after 12 weeks of study, which testifies to the degradation of the amorphous areas of viscose. WAXS studies were also confirmed by Fourier transform infrared spectroscopy (FTIR) and Raman spectroscopy.

Therefore, the mulching material based on viscose nonwoven fabric prepared in the presented article provided the cultivated plants with optimal thermal and water conditions and protected the soil against weed infestation. Moreover, modification of the nonwoven with PLA slowed down the release of mineral fertilizer (KNO_3_) into the growing medium and, at the same time, slowed down the cellulose degradation process. An important feature of the modified nonwoven is its complete biodegradability, which eliminates the problem of disposal of the used crop cover.

## Figures and Tables

**Table 1:** Designations of modified viscose nonwovens.

Type of Nonwoven	Designation of the Nonwoven Sample	Reference Drawing and a SEM Photo of the Nonwoven
Dyed nonwoven	**0**	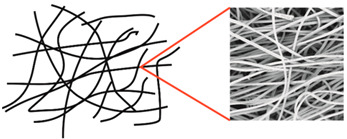
PLA-modified nonwoven	**A**	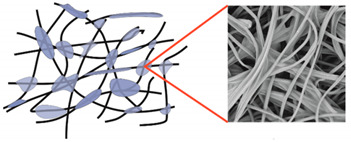
KNO_3_-modified nonwoven	**B**	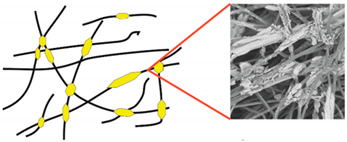
KNO_3_- and PLA-modified nonwoven	**C**	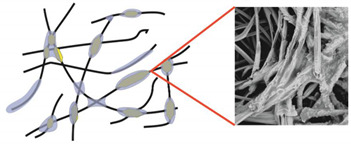

**Table 2:** Physicochemical parameters of the obtained nonwovens.

Designation of the Nonwoven Sample	0	A	B	C
Surface mass (g/m^2^)	55.1 ± 1.8	55.6 ± 0.8	121.8 ± 8.7	126.8 ± 8.9
Air permeability (dm^3^/m^2^/s)	2767 ± 102	2644 ± 39	2443 ± 103	2758 ± 68
Perpendicular water permeability × 10^−3^ (m/s)	87.8 ± 2.0	48.8 ± 6.3	77.0 ± 3.6	72.3 ± 7.2
Breaking force (cN)	857 ± 22	1311 ± 147	1143 ± 31	1148 ± 69
Relative elongation (mm)	11.69 ± 2.00	3.70 ± 0.38	8.89 ± 1.00	7.78 ± 1.00

**Table 3:** The process of nonwoven C biodegradation observed in photographs (a) and SEM micrographs: at a magnification of 1000× (b), and a magnification of 2500× (c). The numbers next to the nonwoven C indicate the weeks of testing.

Designation of the Nonwoven Sample	a	b	c
**0**	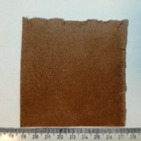	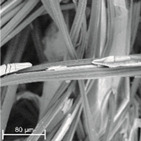	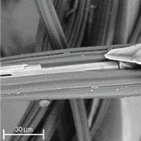
**C2**	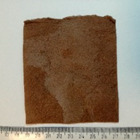	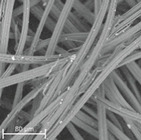	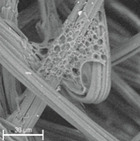
**C4**	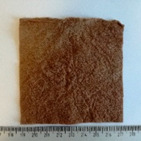	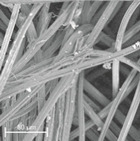	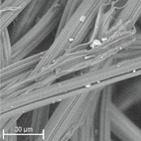
**C6**	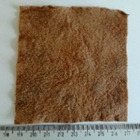	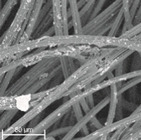	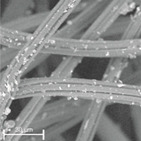
**C8**	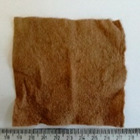	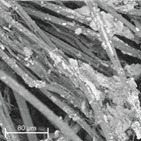	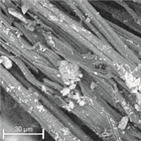
**C10**	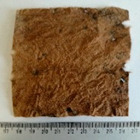	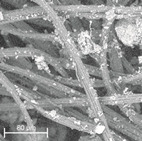	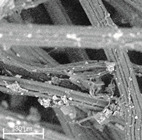
**C12**	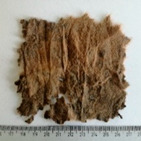	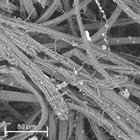	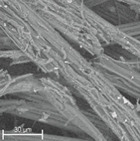

**Table 4:** Physicochemical parameters of nonwoven C during biodegradation studies.

Designation of the Nonwoven Sample	C4	C10	C12	C14
Biodegradation time (weeks)	4	10	12	14
Surface mass (g/m^2^)	77.4 ± 1.4	50.7 ± 4.6	33.0 ± 6.8	15.2 ± 9.9
Air permeability (dm^3^/m^2^/s)	2486 ± 30	1996 ± 58	1644 ± 111	2965 ± 294
Perpendicular water permeability × 10^−3^ (m/s)	76.09 ± 1.65	77.19 ± 0.55	78.29 ± 1.65	177.68 ± 1.92
Breaking force (cN)	820.77 ± 151	311.92 ± 85	270.92 ± 44	187.39 ± 21
Relative elongation (mm)	8.89 ± 3	8.79 ± 1	7.78 ± 1	10.01 ± 2
